# Tuning Genetic Clocks Employing DNA Binding Sites

**DOI:** 10.1371/journal.pone.0041019

**Published:** 2012-07-31

**Authors:** Shridhar Jayanthi, Domitilla Del Vecchio

**Affiliations:** 1 Electrical Engineering and Computer Science, University of Michigan, Ann Arbor, Michigan, United States of America; 2 Mechanical Engineering, Massachusetts Institute of Technology, Cambridge, Massachusetts, United States of America; Tata Institute of Fundamental Research, India

## Abstract

Periodic oscillations play a key role in cell physiology from the cell cycle to circadian clocks. The interplay of positive and negative feedback loops among genes and proteins is ubiquitous in these networks. Often, delays in a negative feedback loop and/or degradation rates are a crucial mechanism to obtain sustained oscillations. How does nature control delays and kinetic rates in feedback networks? Known mechanisms include proper selection of the number of steps composing a feedback loop and alteration of protease activity, respectively. Here, we show that a remarkably simple means to control both delays and effective kinetic rates is the employment of DNA binding sites. We illustrate this design principle on a widely studied activator-repressor clock motif, which is ubiquitous in natural systems. By suitably employing DNA target sites for the activator and/or the repressor, one can switch the clock “on” and “off” and precisely tune its period to a desired value. Our study reveals a design principle to engineer dynamic behavior in biomolecular networks, which may be largely exploited by natural systems and employed for the rational design of synthetic circuits.

## Introduction

**Figure 1:**
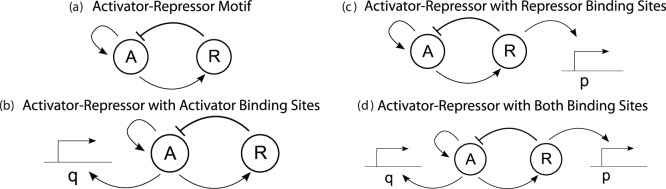
Illustration of the systems analyzed in this paper. Diagram (a) illustrates the activator-repressor motif. Diagram (b) and (c) illustrate the systems after the addition of DNA binding sites with affinity to the activator and the repressor, respectively. Diagram (d) illustrates the case in which both types of DNA binding sites are present.

Periodic oscillations are essential for biological phenomena such as cell cycle regulation and circadian rhythms [1], [2]. Several studies attribute these oscillations to bio-molecular clocks composed of genes arranged in feedback networks [3], [4]. Of the several arrangements that may produce oscillations, *activator-repressor* motifs are recurrent in several natural systems [3], [5]. These motifs comprise an activator module that is self activated and that activates a repressor module. The repressor module, in turn, represses the activator (Figure 1a). This motif has been shown to be remarkably robust to biological noise [5], leading to synthetic implementations as model systems to study natural clocks [6]–[9].

Independently of the specific topology of the network, the presence of delays in feedback loops has long been recognized as a key mechanism to obtain periodic behavior and to tune the clock period (see the review by [1] and the study by [10]). Similarly, a key (related) parameter controlling periodic behavior is the relative value among protein decay rates [11], [12]. For the activator-repressor motif, for example, analytical studies have demonstrated that a crucial mechanism for sustained oscillations is the time-scale difference between the activator and the repressor dynamics, that is, the repressor dynamics should be sufficiently slower than the activator dynamics [13], [14]. This is, to some extent, qualitatively similar to having a delay in the negative feedback from the repressor to the activator. How does nature realize and tune delays and kinetic rates in feedback motifs? Known ways to increase a delay in a feedback or to make the feedback slower include either decreasing the decay rates of species involved in the negative feedback and/or increasing the number of steps in the feedback loop (see, for example, [10], [14], [15]).

Recent studies of modularity in biomolecular circuits have revealed that excess of DNA targets to a protein can slow down the protein’s dynamics [16]–[18]. This effect, called *retroactivity*, is a consequence of changes in the dynamics of the system due to the sequestration of the protein from the network of interactions composing the system. Basically, the protein is “busy” in binding the targets and hence takes longer to perform its function in the system to which it belongs. In the context of modularly designing circuits in synthetic biology, this is an undesired effect (similar to impedance in electrical circuits) that occurs when two modules are interconnected by a transcription factor of one module binding to DNA target sites in the other module. From the perspective of a natural system, however, this loading effect may provide a simple method to tune delays and change the effective kinetic rates without changing the “hardware” of the network.

In this work, we demonstrate that indeed DNA target sites can be employed as a powerful design parameter to finely tune and control the dynamic behavior of a biomolecular circuit, the activator-repressor clock of Figure 1a in particular. Specifically, we illustrate how one can change the dynamics of an activator-repressor clock utilizing DNA binding sites (*load*) with affinity to each of the species. Initially, a mechanism to switch an oscillator “on” or “off” is shown depending on which node (the activator or repressor) the load is being added to. Robustness of this behavior to intrinsic noise is verified by employing stochastic simulation of a mechanistic model of the clock. Finally, a method to tune the period of the clock by employing a carefully chosen amount of load to both nodes is demonstrated.

## Results

We consider a general model for a two-component clock incorporating both positive and negative feedback loops based on the activator-repressor configuration of [6] and illustrated in Figure 1a. Oscillations for activator-repressor clocks often arise from Hopf bifurcation, wherein a stable equilibrium point bifurcates into an unstable equilibrium and a stable periodic orbit when a key parameter is changed [9], [13], [14], [19], [20]. In the models surveyed in the literature, the fundamental mechanism responsible for this oscillatory behavior is well captured by a reduced two-dimensional model that describes the rate of change of the activator and repressor concentrations. This model is obtained by taking into account that the period of oscillations occurs in a timescale slower than the dynamics of multimerization, binding and dissociation interactions, so that quasi-steady state approximations can be made [6], [9], [20]. Additionally, it has been shown that transcription and translation can be lumped into a one-step expression model with no impact to the dynamics of interest [5], [14]. Following these prior works, we also focus on a reduced two-dimensional model.

In the system of Figure 1a, activator protein A promotes its own expression as well as the expression of repressor protein R. Protein R, in turn, represses expression of protein A. Let 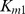 be the apparent dissociation constant between the activator protein and its DNA binding site and 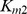 be the apparent dissociation constant between repressor protein and its DNA binding site [21] (see SI for details). For any species X, we denote in italics 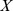 its concentration. Consider the concentration of A and R given in units of their respective dissociation constants 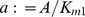 and 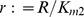. Considering a one-step model for protein expression, the dynamics for this system can be represented by.


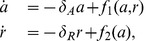
(1)

in which 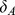 and 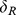 model protein decay (due to either dilution or degradation) and functions 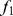 and 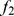 model expression rates and take the form of the standard Hill functions [2]


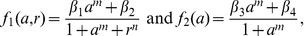
(2)

in which 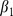 and 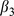 are the maximal expression rates, 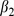 and 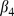 represent the basal expression, and 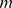 and 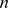 are the Hill coefficients of the affinity between the proteins A and R and their respective binding sites. The mathematical derivation of this reduced nondimensional model is given in the SI. In the sequel, we refer to system (1) as the isolated system.

We assume that the values of the parameters are such that system (1) has a unique equilibrium point. We give conditions for which this assumption holds when either 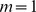 or 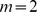 in the SI. In particular, it is shown that when 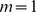, the system always presents a unique and stable equilibrium and, therefore, no oscillatory behavior can be observed. When 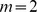 the uniqueness of the equilibrium is guaranteed under the following conditions: (i) the value of 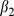 must be sufficiently smaller than the maximal expression rate of the activator, which is proportional to 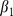; (ii) 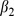 must be non-zero; (iii) the maximal expression rate of the repressor must be larger than the maximal expression rate of the activator; (iv) the smaller 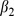 becomes, the smaller 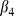 must be. In the general case (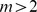), results related to existence and uniqueness of equilibria require a case by case analysis, which is out of the scope of this work. The results from this paper, do not explicitly impose conditions on the Hill coefficients 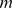 and 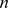 and only assume the uniqueness of the equilibrium 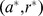 for system (1).

Since system (1) is a two-dimensional system, Poincaré-Bendixson theorem [22] can be employed to obtain conditions for the existence of a periodic orbit. Specifically, one must show that the trajectories of the system are bounded in a compact set and that the equilibrium point is unstable and not locally a saddle.

The following proposition shows that the trajectories of system (1) are bounded in a compact set.

### 

#### Proposition 1

There exists a constant 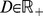 such that the set 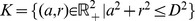 is a positively invariant set under the vector field defined by system (1) and its equilibrium 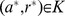.

*Proof.* Note that 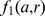 and 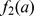 are positive bounded functions in the domain 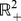. Let 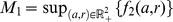 and 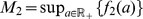. First, notice that for 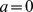, 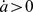 according to (1). Similarly, for 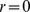, 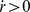. The quadrant 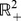 is, therefore, a positively invariant set. Define 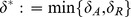 and 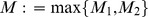. Consider the positive definite function 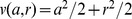. Using the chain rule, we obtain.


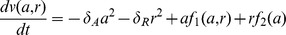



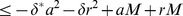



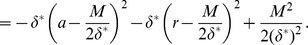


From the above, it is clear that 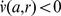 on the exterior of a circle with center 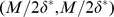 and radius 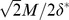. Therefore, for any 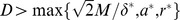, 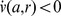 along the arc defined by the boundary of 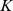. Hence, 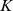 is a positively invariant set that contains the equilibrium 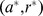.

To show that the equilibrium point is unstable and not locally a saddle, consider the Jacobian matrix of system (1) calculated at the equilibrium:


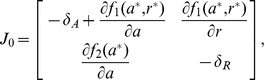
(3)

and denote by 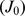 and 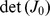 the trace and the determinant of 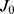, respectively. The eigenvalues of the Jacobian are given by


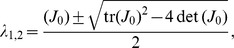


hence the equilibrium point is unstable and not locally a saddle if 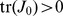 and 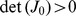. Given the specific expression of the Jacobian in (3), the equilibrium 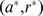 of system (1) is unstable and not locally a saddle if the following conditions are fulfilled:

*(i)* 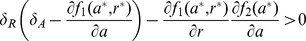 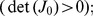


*(ii)* 
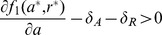
 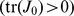


System (1) satisfying conditions *(i)* and *(ii)* presents periodic orbits and will be referred to as *Functional Clock*.

Condition *(ii)* highlights a crucial design principle for the activator-repressor clock. In fact, assume that 
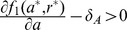
, which is satisfied if the self activation is sufficiently strong. Then, condition *(ii)* can be satisfied if 
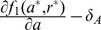
 is sufficiently larger than 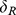. This, in turn, implies that the timescale of the activator dynamics are sufficiently faster than that of the repressor dynamics. Hence, a central mechanism for the appearance of a limit cycle is a fast activator dynamics compared to the repressor dynamics. Retroactivity on a species due to downstream binding sites has been shown to slow down the species dynamics [16], [17]. It follows that downstream binding sites can be employed to vary the relative speeds between the activator and the repressor dynamics. Hence, we will also consider the non-oscillating version of system (1) that does not satisfy condition *(ii)*, referred to as *Non-Functional Clock*. The non-functional clock is given by system (1) in which, in addition to condition *(i)*, the following condition is satisfied:

*(ii)’* 
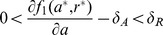
.

**Figure 2:**
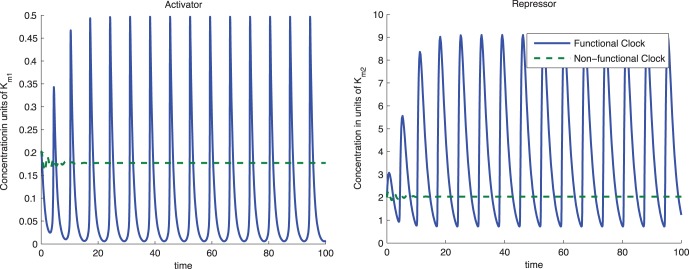
Effect of the trace of the Jacobian on the stability of the equilibrium. The above plots illustrate the trajectories of system (1) for both Functional and Non-Functional Clocks. The parameters in the simulation were 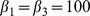, 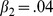, 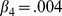 and 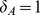. In the Functional Clock, 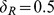 whereas in the Non-Functional Clock, 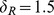. Parameters 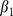 and 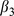 were chosen to give about 500–2000 copies of protein per cell for activated promoters. Parameters 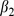 and 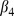 were chosen to give about 1–10 copies per cell for non-activated promoters.

Figure 2 illustrates how conditions *(ii)* and *(ii)’* generate a Functional and a Non-Functional Clock, respectively, by changing the value of parameter 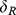.

In this work, we study how the addition of binding sites to the repressor or activator can switch system (1) between the Functional Clock and the Non-Functional Clock behavior, with no change to the parameters of the original system (1).

### Switching the Clock Off by Loading the Activator

In this section, we show the effect of additional DNA binding sites for the activator in a Functional Clock. Specifically, consider system (1) satisfying conditions *(i)* and *(ii)*. The addition of DNA binding sites q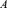 with affinity to the activator A, which binds as homomers, illustrated in Figure 1b, is modeled by the following chemical reaction.


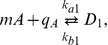
(4)

in which D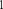 represents the complex formed by A and q. In order to model the addition of DNA binding sites that are identical copies of the ones in the operator, we assume that the affinity between the DNA site q and the activator protein A is given by the apparent dissociation constant 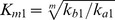, identical to the affinity of A to the promoters in the isolated clock. The impact in the dynamics from retroactivity can be obtained by employing binding sites with different affinities as long as the quantity of binding sites is adjusted accordingly [16]. Additionally, we assume the total concentration of binding sites 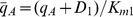 to be constant. Let the complex concentration 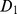 be given in units of 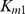 using the nondimensional variable 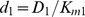. The dynamics of the system after nondimensionalization are given by


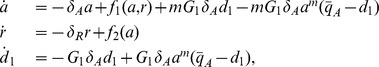
(5)

in which 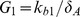 models the timescale separation between the dissociation rate and the protein degradation. A mathematical derivation for this model is found in the SI. Since binding and unbinding reactions can occur in the order of milliseconds, they are in a timescale significantly faster than expression and degradation of proteins, which occur in the order of minutes [2]. As a result, parameter 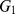 is very large. This fact allows to employ a singular perturbation argument [23], [24] to facilitate the analysis of this system. To this end, define the small parameter 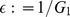 and re-write system (5) as


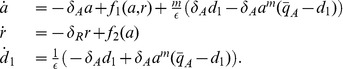
(6)

In order to reduce this system to standard singular perturbation form, we perform the change of variables 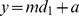, so that system (6) becomes.


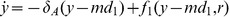
(7)


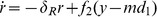
(8)


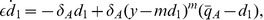
(9)

which is in standard singular perturbation form. Setting 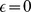 one obtains from (9) the solution 
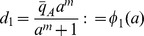
. This equation defines the slow manifold, which can be shown to be locally exponentially stable (see SI). Hence, system (7) is well approximated by the reduced system obtained by replacing 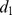 by its expression on the slow manifold 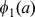. Specifically, we have that


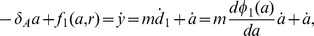


from which we obtain that


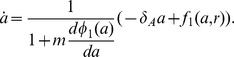


Denoting


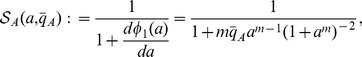


the reduced system in the original coordinates is given by


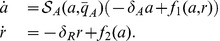
(10)

Since 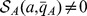, the equilibria of (10) are the same as the ones of (1). Therefore, if (1) has a unique equilibrium 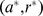, this will also be a unique equilibrium of (10). Also, we have that 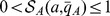 and that 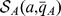 is a strictly monotonically decreasing function of the amounts of DNA binding sites 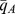. Hence, in system (10), the dynamics of the activator have been slowed down compared to the original isolated system (1). That is, the *effective* kinetic rate of the activator dynamics is now decreased by a factor equal to 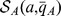. Note additionally that.


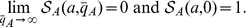
(11)

The Jacobian of system (10) calculated at the equilibrium is given by


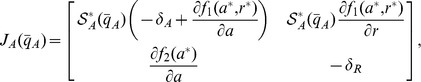
(12)

in which we use the shorthand notation 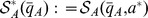. We have 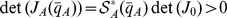 from condition *(i)* and that


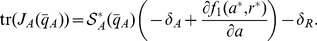


**Figure 3:**
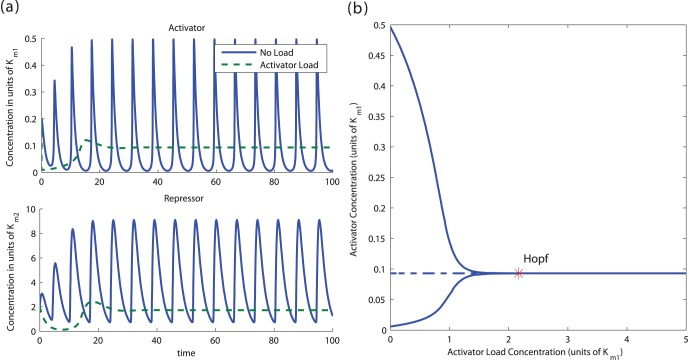
(a) *Load to the Activator can stop a Functional Clock.* The plots illustrate the trajectories of system (5) with two different amounts of load. The parameters in the simulation were 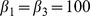, 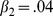, 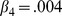, 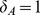, 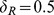, 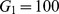, 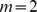 and 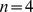. The amount of DNA binding sites in the system with no load is 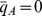 whereas in the system with activator load is 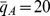. (b) *Bifurcation diagram with load as parameter.* A continuation of the equilibrium as a function of the load parameter 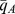 shows that, for this set of parameters, the amount of load to the activator required to stop the clock is on the order of the affinity coefficient 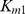, with the bifurcation occurring at 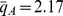. The analysis was made on the full system (5) with the same parameters as before. The solid lines indicate a stable trajectory (the limit cycle to the left side of the Hopf bifurcation point and the equilibrium point to the right side of the Hopf bifurcation point). The dotted line indicates an unstable equilibrium point.

Hence, while the addition of load does not change the sign of the determinant of the Jacobian, it can change the sign of the trace. For large enough load, because of (11), the trace becomes negative and the equilibrium point becomes stable. Hence, the periodic orbit disappears (see the SI for details). Figure 3 a shows the effect of load on system (5).

For the value of 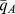 for which 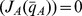, the eigenvalues of the Jacobian are imaginary, hence the system goes through a Hopf bifurcation. A continuation study shows that the Hopf bifurcation is present also in the full three-state system (5). In particular, the amounts of load needed to switch the clock off is about four times the amplitude of the activator oscillations. For the specific choice of parameters in this example, the amount of load required to stop this clock is of the same order of the dissociation constant 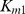, which usually amounts to a low concentration. For example, for the NRI activator used in the oscillator in [25], 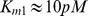 [6] which amounts to approximately 10 copies of the binding site per cell in *E. coli*.

### Switching the Clock on by Loading the Repressor

We now consider a Non-Functional Clock and show how it can be turned into a Functional Clock by adding load to the repressor. Specifically, consider system (1) satisfying conditions *(i)* and *(ii)’*. Following the idea in the previous system, we model here the addition of DNA binding sites q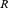 with affinity to the repressor R, identical to the binding sites found in the original clock. This interaction, illustrated in Figure 1c, is modeled by the following chemical reaction.


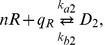
(13)

in which D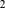 represents the complex formed by the R and q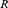. Let the affinity between the repressor and the binding sites is given by the apparent dissociation constant 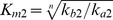. Let 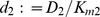 be the nondimensional concentration of complexes and 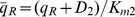 be the total nondimensional concentration of binding sites. The nondimensionalized dynamics of the system are given by


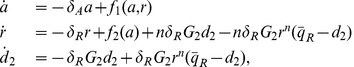
(14)

in which 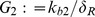 models timescale separation between the dissociation rate of the complex 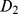 and the repressor decay rate. It is possible to reduce the order of system (14) by a similar technique employed in the previous section. To this end, define 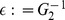. Define also the variable 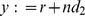, system (14) can be taken to the standard singular perturbation form


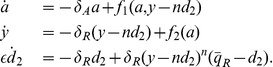
(15)

By setting 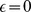, one obtains the reduced system in the original coordinates, which, since the slow manifold is locally exponentially stable (see the SI), is a good approximation of system (14). This reduced system is given by


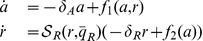
(16)

in which


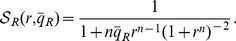


.

Since 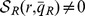, the equilibrium points of (16) are the same as the ones of the isolated system (1). Therefore the unique equilibrium point 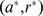 of (1) is also the unique equilibrium point of (16). We employ the shorthand notation 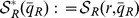. It is easy to verify that 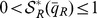 and that 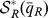 is a strictly monotonically decreasing function of 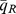. Furthermore, we have that


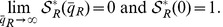
(17)

Hence, the addition of the load to the repressor makes the dynamics of the repressor slower compared to that of the isolated system (1). That is, the repressor *effective* kinetic rates are now smaller by a factor equal to 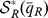, which can be arbitrarily decreased by increasing the amounts of sites 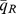. The Jacobian of system (16) calculated at the equilibrium 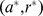 is given by


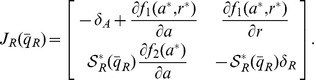
(18)

**Figure 4:**
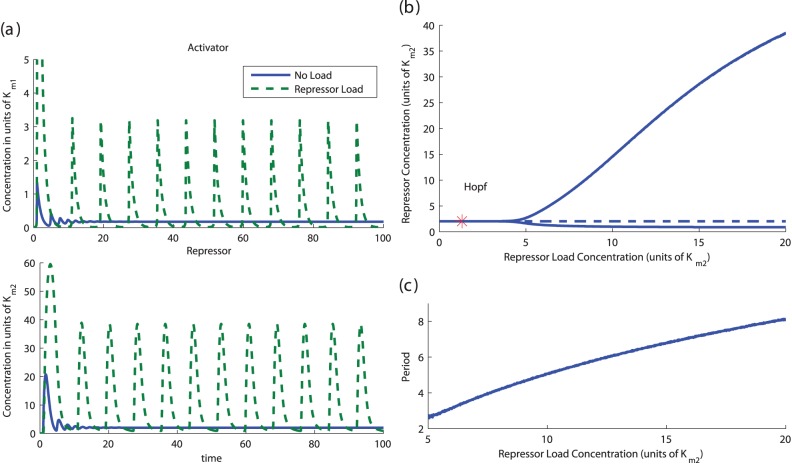
(a) *Load to the Repressor triggers a Non-functional Clock.* The plots illustrate the trajectories of system (14) with two different amounts of load. The parameters in the simulation were 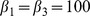, 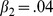, 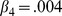, 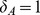, 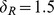, 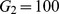, 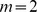 and 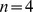. The amount of DNA binding sites in the system with no load is 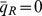 whereas in the system with repressor load is 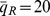. (b) *Hopf Bifurcation with 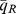 as a parameter.* A continuation of the equilibrium as a function of the load parameter 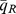 shows that, for this set of parameters, the amount of load required to activate the clock is in the same order of magnitude as that of the the affinity coefficient 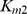, with bifurcation occurring at 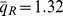. This plot was obtained via continuation of system (14) with the same parameters as before. Solid lines indicate a stable trajectory (limit cycle to the right of the Hopf bifurcation and the equilibrium to its right). The dotted line indicates an unstable equilibrium point. (c) *Period increases as a function of the repressor load 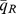.*

Thus, the addition of load to the repressor does not change the sign of the determinant of the Jacobian as 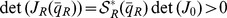. However, it can change the sign of the trace


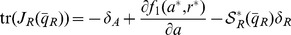


from negative to positive as condition *(ii)’* is satisfied and condition (17) holds. Hence, the equilibrium point can become unstable with sufficient addition of the load and the system begins oscillating (see the formal derivations in the SI). Figure 4a shows the effect of load on system (14). Note that the parameters were chosen so that the system satisfies conditions *(i)* and *(ii)’*.

When 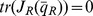, a Hopf bifurcation occurs since both eigenvalues are imaginary. A continuation analysis can be used to show that this Hopf bifurcation is also present in the full system (14). Figure 4b illustrates that the amount of load required for the Hopf bifurcation is given by 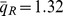 in units of 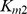. Hence, the amounts of load needed to switch the clock on is on the same order of the amounts of repressor at the equilibrium. For the LacI repressor employed in [6], 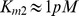 [26], which amounts to few copies per cell of the load.

Figure 4c shows that the addition of load increases the period of oscillation. This suggests the possibility that the load can be employed not only for switching an oscillator “on” and “off” but for also tuning the period. However, the increase in period is accompanied by an increase in the amplitude of the oscillation (Figure 4b), which may be undesired. We discuss how the period can be changed while maintaining the amplitude through simultaneous addition of activator and repressor loads in Section “Tuning the Clock period”.

### Stochastic Simulations of the Switching Behavior

In order to understand how robust the switching behavior is to intrinsic noise, we employ stochastic simulations of the system. An implementation of the Gillespie algorithm [27] was employed to produce realizations of trajectories of an activator repressor clock in which both activator and repressor bind to DNA as dimers (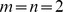).

In these simulations, we assumed the presence of 5 copies of each activator and repressor gene to emulate the situation in which the circuit is present in a low copy number plasmid. Expression rates and degradation rates were chosen based on the values used in the deterministic models to obtain a functional and a non-functional oscillator. The association and dissociation rates between proteins and dimers were chosen so that the apparent dissociation constants 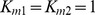, which consider a bacterial transcription factor with apparent dissociation constant on the order of picomolars. A detailed description of this model is given in the SI.

**Figure 5:**
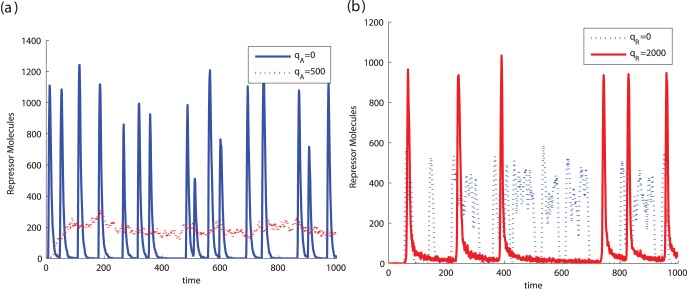
Effect of the load on clock holds under intrinsic noise. The plots above are stochastic realizations of an activator-repressor clock with 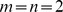 and containing 5 copies of activator and repressor genes. (a) *Functional clock stops with load to the activator.* We show that, with the chosen parameters, it is possible to stop the clock with an amount of load that is roughly 100 times higher than the copy number of the circuit. (b) *Load to the repressor leads to robust oscillation.* We show that, the it is possible to generate robust oscillation with roughly 400 times the number of circuit genes with the choice of parameters above.

Figure 5a shows that addition of binding sites with affinity to the activator can eliminate oscillations from a functional clock. Figure 5b shows how the addition of binding sites with affinity to the repressor can generate sustained more robust oscillations in a non-functional clock. In both situations, the amount of loads employed to switch the clock is on the order of 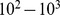 copies of binding sites per cell, which can be achieved by inserting small arrays in high copy number plasmids.

### Tuning the Clock Period

As noticed in Figure 4c, addition of binding sites to the repressor increases the period of the limit cycles of the system. However, this may cause an increase in the amplitude of the cycle (Figure 4b), which may be undesirable. In this section, we illustrate how the simultaneous addition of load to both the activator and repressor can be employed to vary the period as desired with little impact on the cycle amplitude.

Consider the nondimensional model for the system with DNA binding sites for both the activator and the repressor as shown in Figure 1d:
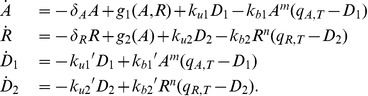
(19)


Here, 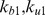 model the association and dissociation rates between the activator protein and its corresponding DNA binding site 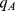, 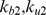 model the association and dissociation rates between the repressor protein and its corresponding DNA binding site 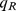, 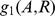, 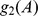 represent the dimensional version of the Hill functions (see SI), and 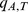, 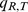 represent the total concentration of activator and repressor DNA sites.

This system can be nondimensionalized, by setting the nondimensional states 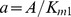, 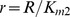, 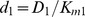 and 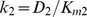, as shown in the SI, to obtain system


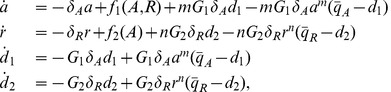
(20)

in which 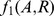 and 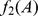 are the nondimensional Hill functions as given in expressions (2), 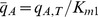 and 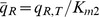, and 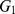 and 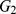 are as defined before. In order to employ a singular perturbation argument similar to what was done in the previous sections, define 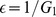, 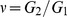 to model the explicit timescale separation present in this system. Define also the following change of variables 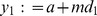 and 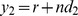. Substituting these in (20), one obtains the system in standard singular perturbation form:


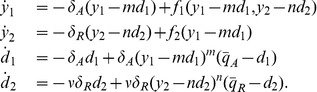
(21)

By setting 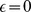, one obtains the slow manifold.


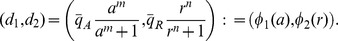


Since the slow manifold is locally exponentially stable (see SI), the reduced system is a good approximation of system (21). Since 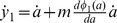 and 
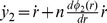
, this reduced system, in the original variables, takes the form


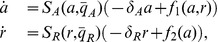
(22)

in which


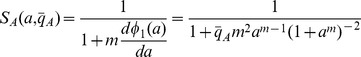


and


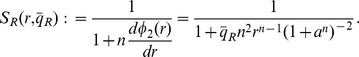


Let the activator and repressor loads be added at a fixed ratio 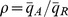 and define 
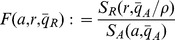
. System (22) can be re-written as


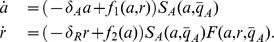
(23)

Since 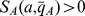, this system is orbitally equivalent [19] to the system.


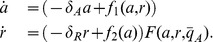
(24)

Hence, if system (23) has a periodic orbit, system (24) will have a corresponding periodic orbit with identical trajectories. The corresponding periodic signals, however, will have different periods whose values depend on function 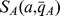. Thus, if the value of 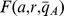 does not appreciably change when 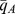 changes, the addition of the load will affect the period of oscillations without impacting their amplitudes. Since.


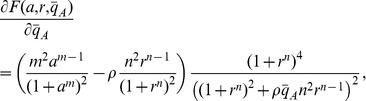
(25)

we have that for large values of 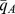, 
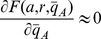
. Under these conditions, since the function 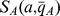 is a monotonically decreasing function of 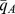, the periodic orbits of system (23) will display decreasing periods as 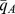 increases, while maintaining the same amplitude, due to orbital equivalence between system (24) and system (23) (see the SI for a formal proof).

**Figure 6:**
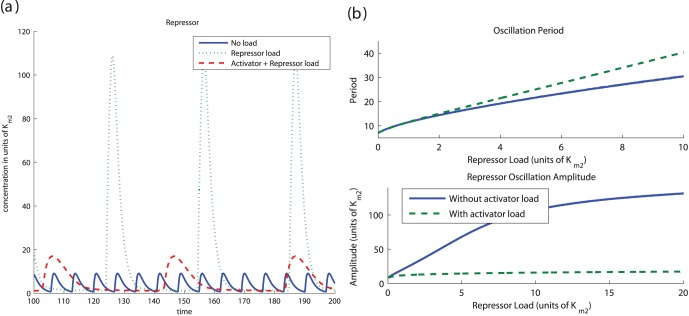
Tuning the period without affecting the amplitude. (a) When compared to the isolated system, the amplitude of oscillations in system (20) increases when we add exclusively DNA binding sites with affinity to the repressor (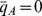, 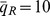). However, if we simultaneously add DNA binding sites with affinity to the activator, the amplitude is not affected as much (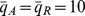). (b) The period of system (20) can be changed with no effect on the amplitude when DNA binding sites with affinity to both the repressor and the activator are added simultaneously. The upper plot shows that a similar increase of period observed via the addition of repressor load can be obtained via the simultaneous addition of activator and repressor load. This second method has the advantage of not generating an increase in the amplitude, as shown in the lower plot. In this simulation we assumed the ratio 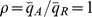. Parameters of the activator repressor system used in the simulation were 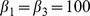, 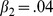, 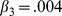, 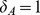, 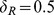, 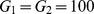 and 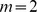, 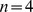. In the traces showing only repressor load 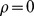, while the traces showing simultaneous repressor and activator load, 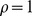.

Figure 6a illustrates this result. The addition of repressor load to a functioning clock increases the period but also leads to a higher amplitude. This effect in the amplitude is not observed when both activator and repressor loads are added. Figure 6b shows this behavior for increasing amount of load. When only repressor load is added, there is an increase in the period of the limit cycles along with an increase in the amplitude, as it was seen in the previous section (Figure 4(b) and (c)). However, if a sufficient amount of activator load is simultaneously added along with the repressor load, the increase of the period occurs with very little impact on the amplitude of oscillations.

## Discussion

Effective kinetic rates are crucial parameters for the dynamic behavior of biomolecular networks. In particular, delays in negative feedback loops have been shown to be a fundamental mechanism for periodic oscillations both in electronic circuits [28] and in biomolecular networks [1], [10], [15]. Research has shown that in natural systems these delays are realized by the number of steps, such as transcription, translation, and post-translational modifications, involved in the implementation of the feedback loop. More steps lead to larger delays. Hence, adding a delay involves engineering the structure and length of a pathway. In this paper, we have revealed that a different mechanism exists for adding and carefully tuning delays and effective kinetic rates: the addition of DNA targets. In natural systems, transcription factors can have large numbers of DNA binding sites, several of which do not even have regulatory functions (see [29] and [30], for example). Our study suggests that a role of these DNA binding sites is to carefully tune effective kinetic rates to realize the desired dynamics in genetic networks.

As an example, consider the regulation network of cellular resources such as ribosomes or RNA polymerase (RNAP). Since both molecules need themselves to be assembled, there is a self activating loop. Additionally, it has been shown [31], [32] that RNAP and ribosomes are negatively regulated through transcriptional repression. Hence, the regulation motif of these species has the form of Figure 1a, in which we can view A as the resource (RNAP or ribosome) and R as a repressor system. This motif, as we have shown, can present sustained oscillations, which would be undesired for RNAP or ribosomes. However, due to the large demand by the cellular environment, RNAP and ribosomes are being used through (reversible) binding processes, so that the actual motif is closer to that of Figure 1(b). Since the amount of q is fairly high, the system is brought back to stability.

The capacity of tuning the clock stems from the sequestration effect of the protein by DNA binding sites, similarly to [33]. More generally, this effect can be achieved by employing various protein loads, such as substrates, inhibitors [20], and targets on other proteins [17], [34], [35]. It has been shown that sequestration can be used to tune a synthetic bio-molecular circuit by changing steady-state characteristics such as sensitivity [35] or steady-state level [33]. In contrast, the result in this work comes from an impact in the dynamical behavior. The model employed in this work assumes implicitly that the DNA sites protect the transcription factors, and therefore the steady-state characteristics of free protein is unaffected by the sequestration [30]. However, in spite of the increased amounts of total protein, the additional DNA sites are still able to slow down the dynamics by increasing the demand for the transcription factor. In other words, the protein becomes “busy” having to interact with additional DNA sites.

The mechanism revealed in this paper for tuning effective kinetic rates is especially relevant for synthetic circuits due to its simple implementation. Instead of modifying promoter or operator regions, or changing degradation tags or protease recognition motifs, a simple addition of DNA with binding sites through transformation. or transfection can achieve the desired modifications. This can also simplify prototyping of some synthetic biology modules whose correct function is sensitive to specific kinetic parameters. Gradual addition of DNA sites through inducible plasmids, for example, could be employed to search the parameter space for expected behavior before final adjustment of expression, degradation, and dissociation constants.

## Materials and Methods

Simulations were performed using the ode23 s numerical solver that comes in MATLAB. Continuation diagrams were made using Matcont. Stochastic simulations were made using an implementation of the Stochastic Simulation Algorithm as described in [27] in a C/POSIX environment. The parameters used in all deterministic simulations are shown in the captions of the figures. Parameters used in the stochastic simulations are given in the Information S1.

## Supporting Information

Information S1 **The Supporting Information file contains technical aspects of the results in this work that were not included in the manuscript to preserve text clarity.** Specifically the file contains (S1) the Hill functions employed; (S2) nondimensionalization of the model; (S3) conditions for stability of the equilibrium of the oscilator; (S4) proof of the effects of the load on the stability of the equilibrium; (S5) proof of the stability of the slow manifold required for application of the singular perturbation technique; and (S6) a demonstration of orbital equivalence. The SI also contains (S7) the mechanistic model employed for Stochastic Simulation.
(PDF)
