# Supplementary material for: Tuning Genetic Clocks Employing DNA Binding Sites
Source: PLoS One. 2012 Jul 31;7(7):e41019. doi: 10.1371/journal.pone.0041019 (PMC3409220; doi:10.1371/journal.pone.0041019)
Supplement: Information S1 — The Supporting Information file contains technical aspects of the results in this work that were not included in the manuscript to preserve text clarity. Specifically the file contains (S1) the Hill functions employed; (S2) nondimensionalization of the model; (S3) conditions for stability of the equilibrium of the oscilator; (S4) proof of the effects of the load on the stability of the equilibrium; (S5) proof of the stability of the slow manifold required for application of the singular perturbation technique; and (S6) a demonstration of orbital equivalence. The SI also contains (S7) the mechanistic model employed for Stochastic Simulation. (PDF) [file pone.0041019.s001.pdf]

Supporting Information
"Tuning Genetic Clocks Employing DNA Binding Sites"

Shridhar Jayanthi[1,2] and Domitilla Del Vecchio[2*]

1 Electrical Engineering and Computer Science, University of Michigan, Ann Arbor, MI, USA

2 Mechanical Engineering, Massachusetts Institute of Technology, Cambridge, MA, USA

∗ **E-mail: ddv@mit.edu**

S1 Model of Hill functions

In this section we identify the Hill function approximations for the expression of proteins controlled by *(i)* an activator protein and *(ii)* a repressor and an activator protein. Consider first the expression of protein X whose expression rate is regulated by an activator protein A via the promoter p_R. These processes can be modeled by the following chemical reactions

$$\begin{aligned} p_R + mA &\underset{k_{b1}}{\overset{k_{a1}}{\rightleftharpoons}} C_1 \\ C_1 &\xrightarrow{\kappa_2} C_1 + X \\ p_R &\xrightarrow{\kappa_4} p_R + X, \end{aligned} \tag{S1}$$

in which κ_2 is the expression level of the promoter bound to A, κ_4 is the basal expression level of the promoter, k_{a1} and k_{b1} are the association and dissociation rates of the promoter to A respectively and m models the cooperative binding of the activator protein. Assuming that there is a conservation of the total amount of promoter sites, modeled by the expression $p_R + C_1 = p_{R,T}$, the expression level from this promoter can be modeled by $g_2(A) = \kappa_2 C_1(A) + \kappa_4(p_{R,T} - C_1(A))$. The quasi-steady state value of C_1 can be obtained by identifying the equilibrium of the following ODE

$$\dot{C}_1 = k_{a1}(p_{R,T} - C_1)A^m - k_{b1}C_1. \tag{S2}$$

Defining $K_{m1} = \sqrt[m]{k_{b1}/k_{a1}}$, we obtain

$$g_2(A) = \kappa_2 p_{R,T} \frac{A^m}{A^m + K_{m1}^m} + \kappa_4 p_{R,T} \frac{K_{m1}^m}{A^m + K_{m1}^m} = \frac{K_2 A^m + K_4 K_{m1}^m}{A^m + K_{m1}^m}, \tag{S3}$$

in which $K_2 := \kappa_2 p_{R,T}$ and $K_4 := \kappa_4 p_{R,T}$.

Consider now the expression of a protein X whose expression rate is regulated by an activator protein A as well as by repressor protein R via the promoter p_A. We will assume that the binding is competitive. Expression can be modeled by the following chemical reactions

$$\begin{aligned} p_A + mA &\underset{k_{b1}}{\overset{k_{a1}}{\rightleftharpoons}} C_1 \\ p_A + nR &\underset{k_{b2}}{\overset{k_{a2}}{\rightleftharpoons}} C_2 \\ C_1 &\xrightarrow{\kappa_1} C_1 + X \\ p_A &\xrightarrow{\kappa_3} p_A + X, \end{aligned} \tag{S4}$$

in which κ_1 is the expression level of the promoter bound to A, κ_3 is the basal expression level of the promoter, k_{a1} and k_{b1} are the association and dissociation rates of the promoter to A, respectively, k_{a2} and k_{b2} are the association and dissociation rates of the promoter to R, respectively, and m and n model the cooperative binding of the activator and repressor proteins, respectively. We assume that the repressor activity is perfect and therefore no expression can occur from the repressed promoter. Assuming that there is a conservation of the total amount of promoter sites, modeled by the expression $p_A + C_1 + C_2 = p_{A,T}$, the expression level from this promoter can be modeled by $g_1(A,R) = \kappa_1 C_1(A) + \kappa_3(p_{A,T} - C_1(A) - C_2(R))$. The quasi-steady state value of C_1 and C_2 can be obtained by identifying the equilibrium of the following ODE

$$\begin{aligned} \dot{C}_1 &= k_{a1}(p_{A,T} - C_1 - C_2)A^m - k_{b1}C_1 \\ \dot{C}_2 &= k_{a2}(p_{A,T} - C_1 - C_2)R^n - k_{b2}C_2 \end{aligned} \tag{S5}$$

Defining $K_{m1} = (k_{b1}/k_{a1})^{1/m}$ and $K_{m2} = (k_{b2}/k_{a2})^{1/n}$, we obtain the expression

$$g_1(A,R) = p_{A,T} \frac{\kappa_1 K_{m2}^n A^m + \kappa_3 K_{m1}^m K_{m2}^n}{K_{m1}^m K_{m2}^n + K_{m2}^n A^m + K_{m1}^m R^n} = \frac{K_1 K_{m2}^n A^m + K_3 K_{m1}^m K_{m2}^n}{K_{m1}^m K_{m2}^n + K_{m2}^n A^m + K_{m1}^m R^n}, \tag{S6}$$

in which $K_1 := \kappa_1 p_{A,T}$ and $K_3 := \kappa_3 p_{A,T}$.

S2 Nondimensionalization of the activator repressor clock

In this section, we identify a nondimensional model of the activator repressor clock having loads to activator and repressor, given in Figure 1d. The association and dissociation between transcription factor A and R and their respective additional binding sites q_A and q_R are model by the following dynamics

$$q_A + mA \underset{k'_{b1}}{\overset{k'_{a1}}{\rightleftharpoons}} D_1 \tag{S7}$$

$$q_R + nR \underset{k'_{b2}}{\overset{k'_{a2}}{\rightleftharpoons}} D_2. \tag{S8}$$

The model for this system can be obtained by adding the binding dynamics to the model given in [14] for the activator-repressor clock as

$$\begin{aligned} \dot{A} &= -\delta_A A + g_1(A,R) + mk'_{b1}D_1 - mk'_{a1}A^m(q_{A,T} - D_1) \\ \dot{R} &= -\delta_R R + g_2(A) + nk'_{b2}D_2 - nk'_{a2}R^n(q_R - D_2) \\ \dot{D}_1 &= -k'_{b1}D_1 + k'_{a1}A^m(q_{A,T} - D_1) \\ \dot{D}_2 &= -k'_{b2}D_2 + k'_{a2}R^n(q_{R,T} - D_2), \end{aligned} \tag{S9}$$

in which $q_{A,T} := q_A + D_1$ and $q_{R,T} := q_R + D_2$ model the total amount of DNA bindings sites in the system, δ_A and δ_R model protein decay (due to either dilution or degradation) and functions f_1 and f_2 model expression rates and take the form of the standard Hill functions derived on Section S1.

$$g_1(A,R) = \frac{K_1(A/K_{m1})^m + K_3}{1 + (A/K_{m1})^m + (R/K_{m2})^n} \text{ and } g_2(A) = \frac{K_2(A/K_{m1})^m + K_4}{1 + (A/K_{m1})^m}, \tag{S10}$$

in which K_1 and K_2 are the maximal expression rates, K_3 and K_4 represent the basal expression, K_{m1} and K_{m2} is related to the affinity between the proteins and their respective binding sites and m and n are the Hill coefficients related to the multimerization of activator and repressor proteins, respectively. Define $G_1 := k'_{b1}/\delta_A$ and $G_2 := k'_{b2}/\delta_R$ to be non-dimensional constants modeling the timescale difference between complex dissociation and transcription factor degradations rates. Define additionally $K'_{m1} := \sqrt[m]{k'_{b1}/k'_{a1}}$ and $K'_{m2} = \sqrt[n]{k'_{b2}/k'_{a2}}$ as the apparent dissociation constant as defined

in [21].

From this system, define the nondimensional variables $a := A/K_{m1}$, $r := R/K_{m2}$, $d = D_1/K'_{m1}$ and $d_2 = D_2/K'_{m2}$. Let $\sigma_1 = K'_{m1}/K_{m1}$ and let $\sigma_2 = K'_{m2}/K_{m2}$ describe the difference in affinity of the transcription factor to the promoter in the circuit or the additional DNA load. The differential equation is then reduced to

$$\begin{aligned}
\dot{a} &= -\delta_A a + \frac{\beta_1 a^m + \beta_2}{1 + a^m + r^n} + mG_1\delta_A\sigma_1 d_1 - mG_1\delta_A\sigma_1^{(1-m)} a^m(\bar{q}_A - d_1) \\
\dot{r} &= -\delta_R r + \frac{\beta_3 a^m + \beta_4}{1 + a^m} + nG_2\delta_R\sigma_2 d_2 - nG_2\delta_R\sigma_2^{(1-n)} r^n(\bar{q}_R - d_2) \\
\dot{d}_1 &= -G_1\delta_A d_1 + G_1\delta_A\sigma_1^{-m} a^m(\bar{q}_A - d_1) \\
\dot{d}_2 &= -G_2\delta_R d_2 + G_2\delta_R\sigma_2^{-n} r^n(\bar{q}_R - d_2),
\end{aligned} \tag{S11}$$

in which $\beta_1 := K_1/K_{m1}$, $\beta_2 := K_A/K_{m1}$, $\beta_3 := K_2/K_{m2}$, $\beta_4 := K_R/K_{m2}$, $\bar{q}_A = q_{A,T}/K'_{m1}$ and $\bar{q}_R = q_{R,T}/K'_{m2}$.

From system (S11), one can obtain non-dimensional models for the various systems described in this paper. In particular, to obtain (1), $\bar{q}_R = \bar{q}_A = 0$; in (5) $\bar{q}_R = 0$ and $\sigma_1 = 1$; in (14) $\bar{q}_A = 0$ and $\sigma_2 = 1$ and finally in (20) $\sigma_1 = \sigma_2 = 1$.

S3 Conditions for a unique and unstable equilibrium

We next establish parameter conditions for which we can guarantee that there is a unique equilibrium of system (1).

Let $\bar{\beta}_1 = \beta_1/\delta_A$, $\bar{\beta}_2 = \beta_2/\delta_A$, $\bar{\beta}_3 = \beta_3/\delta_R$, $\bar{\beta}_4 = \beta_4/\delta_R$ and let

$$f(a, r) := -\delta_A a + f_1(a, r) \text{ and } g(a, r) := -\delta_R r + f_2(a). \tag{S12}$$

Then, the nullclines are given by $f(a, r) = 0$ and $g(a, r) = 0$, which define r as a function of a in the following way:

$$f(a, r) = 0 \quad \Longrightarrow \quad r = \left(\frac{\bar{\beta}_1 a^m + \bar{\beta}_2 - a(1 + a^m)}{a}\right)^{1/n} \tag{S13}$$

$$g(a, r) = 0 \quad \Longrightarrow \quad r = \frac{\bar{\beta}_3 a^m + \bar{\beta}_4}{1 + a^m}. \tag{S14}$$

Proposition 2. *If $m = 1$, system (1) admits a unique stable equilibrium point. If $m = 2$, system (1) admits a unique unstable (not locally a saddle) equilibrium point if the following parameter relations are verified*

$$0 < \bar{\beta}_2 \leq \frac{\bar{\beta}_1^3}{27},\ L \leq \frac{\bar{\beta}_3 A_L^2 + \bar{\beta}_4}{1 + A_L^2},\ l \geq \frac{\bar{\beta}_3 A_l^2 + \bar{\beta}_4}{1 + A_l^2}, \tag{S15}$$

and

$$\left.\frac{\delta_R}{\partial f_1/\partial a}\right|_{(a^*,r^*)} - \delta_A < 1, \tag{S16}$$

in which

$$\begin{aligned} A_l &= \frac{\bar{\beta}_1}{6}\left(1 - (cos(\phi/3) - \sqrt{3}sin(\phi/3))\right) \\ A_L &= \frac{\bar{\beta}_1}{6} + \frac{\bar{\beta}_1}{3}cos(\phi/3) \\ \phi &= atan\left(\frac{\sqrt{27\bar{\beta}_2(\bar{\beta}_1^3 - 27\bar{\beta}_2)}}{\frac{\bar{\beta}_1^3}{2} - 27\bar{\beta}_2}\right), \end{aligned} \tag{S17}$$

$$l = \sqrt[n]{\frac{\bar{\beta}_1 A_l^2 + \bar{\beta}_2 - A_l(1 + A_l^2)}{A_l}},$$

$$L = \sqrt[n]{\frac{\bar{\beta}_1 A_L^2 + \bar{\beta}_2 - A_L(1 + A_L^2)}{A_L}}.$$

Proof. The Jacobian at $S^* := (a^*, r^*)$ is given by the matrix

$$J(S^*) = \begin{pmatrix} \frac{\partial f}{\partial a} & \frac{\partial f}{\partial r} \\ \frac{\partial g}{\partial a} & \frac{\partial g}{\partial r}, \end{pmatrix},$$

in which the partial derivatives are computed at the equilibrium point S^*. For an unstable node or spiral to occur, it is sufficient that

$$(i)\ \mathrm{tr}(J(S^*)) > 0 \text{ and } (ii)\ \det(J(S^*)) > 0.$$

Case 1: $m = 1$. The nullcline $f(a, r) = 0$ has always negative slope, and therefore we always have only

one equilibrium point. Furthermore, expression (S13) with $m = 1$ leads to

$$\left.\frac{dr}{da}\right|_{f(a,r)=0} = -\frac{r^{-1+1/n}}{n}\frac{a^2+\bar{\beta}_2}{a^2} < 0.$$

Since $dr/da|_{f(a,r)=0} = -(\partial f/\partial a)/(\partial f/\partial r)$ by the implicit function theorem and since $\partial f/\partial r < 0$, it must be that $\partial f/\partial r < 0$. As a consequence, $\mathrm{tr}(J(S^*)) < 0$ because $\frac{\partial g}{\partial r} = -\delta_R < 0$. To show that both eigenvalues of $J(S^*)$ are negative, we are left to show that $\det(J(S^*)) > 0$. This is readily seen to be true as we have that

$$\left.\frac{dr}{da}\right|_{g(a,r)=0} = -\frac{\partial g/\partial a}{\partial g/\partial r} > \left.\frac{dr}{da}\right|_{f(a,r)=0} = -\frac{\partial f/\partial a}{\partial f/\partial r} < 0,$$

thus implying that $\frac{\partial f}{\partial a}\frac{\partial g}{\partial r} - \frac{\partial f}{\partial r}\frac{\partial g}{\partial a} = \det(J(S^*)) > 0$.

Case 2: $m = 2$. Figure S1 shows the only possible configuration of the nullclines in which (a) we have a unique equilibrium and (b) the nullclines are intersecting with the same positive slope. The plots imply that

$$\frac{dr}{da}\Big|_{g(a,r)=0} = -\frac{\partial g/\partial a}{\partial g/\partial r} > \frac{dr}{da}\Big|_{f(a,r)=0} = -\frac{\partial f/\partial a}{\partial f/\partial r} > 0,$$

and thus that $\frac{\partial f}{\partial a}\frac{\partial g}{\partial r} - \frac{\partial f}{\partial r}\frac{\partial g}{\partial a} = \det(J(S^*)) > 0$. By relations (S12), we have that $\partial g/\partial a = \partial f_2/\partial a$, $\partial g/\partial r = -\delta_R$, $\partial f/\partial a = (-\delta_A + \partial f_1/\partial a)$, and $\partial f/\partial r = -|\partial f_1/\partial r|$. If at the equilibrium point S^* the nullcline $f(a, r) = 0$ has negative slope, S^* is stable, as we have shown for the case $m = 1$. Therefore, we examine what additional conditions should be enforced to guarantee that the equilibrium point is unstable when the nullclines intersect both with positive slopes. Since condition (ii) is verified by the condition that the nullclines cross with positive slopes, we are left to provide conditions for which (i) is also true. To have that $\mathrm{tr}(J(S^*)) > 0$, we require that $(\frac{\partial f_1}{\partial a} - \delta_A) - \delta_R > 0$, which is verified if condition (S16) holds.

We finally determine sufficient conditions on the parameters for having one crossing and such that the slopes of the two nullclines at the crossing are both positive (and thus (ii) is verified). This is performed by simple geometric considerations. For this purpose, consider Figure S1.

The values A_l and A_L of the location of the minimum and maximum of $f(a, r) = 0$ can be computed by computing the derivative with respect to A of expression

$$r^n = \frac{\bar{\beta}_1 a^2 + \bar{\beta}_2 - a(1 + a^2)}{a}$$

obtained by (S13) and equating it to zero, as the square root function is monotone. This way, we find a third order polynomial that has two positive roots if $0 < \bar{\beta}_2 \leq \frac{\bar{\beta}_1^3}{27}$, otherwise it has one positive and two complex roots. These roots are given by relations (S17) and they are shown in Figure S1. Thus, by looking at the same figure, one deduces that if conditions (S15) are satisfied, we have on equilibrium point only, and (ii) is verified. □

For having one equilibrium point only, we require the activator basal transcription rate, proportional to $\bar{\beta}_2$, to be sufficiently smaller then the maximal expression rate of the activator, which is proportional to $\bar{\beta}_1$. Also, $\bar{\beta}_2$ must be non-zero. Also, in case $\bar{\beta}_1 >> \bar{\beta}_2$, one can verify that $A_L \approx \bar{\beta}_1/2$ and thus $L \approx \sqrt[n]{\bar{\beta}_1^2/4}$. As a consequence, conditions (S15) require also that if $\bar{\beta}_1$ increases then so must do $\bar{\beta}_3$. This qualitatively implies that the maximal expression rate of the repressor must be larger than the maximal expression rate of the activator, when expressed in units of the affinity constant. Finally, $A_l \approx 0$ and $l \approx \sqrt[n]{\bar{\beta}_2/A_l}$. As a consequence, conditions (S15) also imply that the smaller $\bar{\beta}_2$ becomes, the smaller $\bar{\beta}_3$ must be.

S4 Proofs on the effect of load

Proposition 3. *Consider system (10) satisfying conditions (i) and (ii). There exists $q^* > 0$ such that the equilibrium (a^*, r^*) is asymptotically stable if and only if $\bar{q}_A > q^*$.*

Proof. We first show that $\det(J_A(\bar{q}_A)) > 0$ for all $\bar{q}_A$. This follows from the fact that $\det(J_A(\bar{q}_A)) = \mathcal{S}_A^*(\bar{q}_A)\det(J_0) > 0$, from condition *(i)*. We now focus on

$$\mathrm{tr}(J_A(\bar{q}_A)) = \mathcal{S}_A^*(\bar{q}_A)\left[-\delta_a + \frac{\partial f_1(a^*, r^*)}{\partial a}\right] - \delta_R.$$

From (11) and condition *(ii)*, when $\bar{q}_A = 0$ $\mathrm{tr}(J_A(0)) > 0$. Additionally, as $\bar{q}_A \to \infty$, $\mathrm{tr}(J_A(\bar{q}_A)) \to -\delta_R < 0$. Since the trace is a monotonic smooth function of $\bar{q}_A$, one can apply the intermediate value theorem to show that there is an unique $0 < q^* < \infty$ such that $\mathrm{tr}(J_A(q^*)) = 0$. Since $\det(J_A(q^*)) > 0$, the eigenvalues of $J_A(q^*)$ are imaginary. From the monotonicity of the trace with respect to $\bar{q}_A$, it follows that the real parts of the eigenvalues of $J_A(\bar{q}_A)$ are positive for all $0 \leq \bar{q}_A < q^*$ and negative for all $\bar{q}_A > q^*$. It follows that the system goes through a Hopf bifurcation at $\bar{q}_A = q^*$, and thus presents a periodic solution for $0 \leq q_A < q^*$ while it converges to the equilibrium for $\bar{q}_A > q^*$. □

Proposition 4. *Consider system (16) satisfying conditions (i) and (ii)'. There exists a* $q^* > 0$ *such that the equilibrium* (a^*, r^*) *is asymptotically stable if and only if* $\bar{q}_R < q^*$.

Proof. We first show that the $\det(J_R(\bar{q}_R)) > 0$ for all q_R. This follows from the fact that $\det(J_R(\bar{q}_R)) = \mathcal{S}_R^*(\bar{q}_R)\det(J_0) > 0$ from condition *(i)*. We now proceed to show that the trace can change its sign. Note that

$$\mathrm{tr}(J_R(\bar{q}_R)) = -\delta_A + \frac{\partial f_1(a^*, r^*)}{\partial a} - \mathcal{S}_R^*(\bar{q}_R)\delta_R.$$

From (17) and condition *(ii)'*, when $\bar{q}_R = 0$, $\mathrm{tr}(J_R(\bar{q}_R)) < 0$. Additionally, as $lim_{\bar{q}_R \to \infty} \mathrm{tr}(J_R(\bar{q}_R)) = -\delta_A + \frac{\partial f_1(a^*, r^*)}{\partial a} < 0$ from condition *(ii)'*. Since the trace is a monotonic smooth function of $\bar{q}_R$, one can apply the intermediate value theorem to show that there is an unique $0 < q^* < \infty$ such that $\mathrm{tr}\, J_R(q^*) = 0$. Since $\det(J_R(q^*)) > 0$, the eigenvalues of $J_R(q^*)$ are imaginary. From the monotonicity of the trace with respect to $\bar{q}_R$, it follows that the real parts of the eigenvalues of $J_R(\bar{q}_R)$ are negative for all $0 \leq \bar{q}_R < q^*$ and positive for all $q_R > q^*$. It follows thus that the system goes through a Hopf bifurcation at $\bar{q}_R = q^*$ and thus presents a periodic solution for $\bar{q}_R > q^*$ while it converges to the equilibrium for $\bar{q}_R < q^*$. □

S5 Proofs on stability of the slow manifolds

Proposition 5. *The stability of the slow manifold* $d_1 = \psi_1(y)$ *defined by setting* $\epsilon = 0$ *in system (7-9) is locally exponentially stable.*

Proof. The manifold $d_1 = \psi_1(y)$ is the unique solution of the algebraic equation

$$g(y, d_1) := -\delta_A d_1 + \delta_A (y - m d_1)^m (q_T - d_1) = 0.$$

Note that, since $0 \leq d_1 \leq q_T$, $0 \leq \psi_1(y) \leq q_T$.

To prove this proposition, we need to show that $\left.\frac{\partial g(y, d_1)}{\partial d_1}\right|_{d_1 = \psi_1(y)} < 0$ [23].

$$\frac{\partial g(y, d_1)}{\partial d_1} = -\delta_A - m\delta_A (y - m d_1)^{m-1}(\bar{q}_A - d_1) - \delta_A (y - m d_1)^m.$$

Since $g(y,\psi_1(y)) = 0$, $y - m\psi_1(y) = \sqrt[m]{\dfrac{\psi_1(y)}{\bar{q}_A - \psi_1(y)}}$ and therefore

$$\left.\frac{\partial g(y,d_1)}{\partial d_1}\right|_{d_1=\psi_1(y)} = -\delta_A - m\delta_A \left(\frac{\psi_1(y)}{\bar{q}_A - \psi_1(y)}\right)^{\frac{m-1}{m}} (\bar{q}_A - \psi_1(y)) - \delta_A \frac{\psi_1(y)}{\bar{q}_A - \psi_1(y)} < 0,$$

since $0 \le \psi_1(y) \le \bar{q}_A$ for all values of y as shown above. □

Proposition 6. *The stability of the manifold $d_2 = \psi_2(y)$ defined by setting $\epsilon = 0$ in system (15) is locally exponentially stable.*

Proof. The proof of this result is similar to the proof of the previous proposition. Here we must show that $\left.\dfrac{\partial h(y,d_2)}{\partial d_2}\right|_{d_2=\psi_2(y)} < 0$ where the manifold $d_2 = \psi_2(y)$ is the unique solution of equation

$$h(y,d_2) := -\delta_R d_2 + \delta_R (y - nd_2)^n (\bar{q}_R - d_2) = 0.$$

Since $0 \le d_2 \le \bar{q}_R$, $0 \le \psi_2(y) \le \bar{q}_R$. Additionally, from the definition of the manifold, $y - n\psi_2(y) = \sqrt[n]{\dfrac{\psi_2(y)}{q + R - \psi_2(y)}}$. Therefore

$$\begin{aligned}\left.\frac{\partial h(y,d_2)}{\partial d_2}\right|_{d_2=\psi_2(y)} &= -\delta_R - n\delta_R (y - n\psi_2(y))^{n-1} (\bar{q}_R - \psi_2(y)) - \delta_R (y - n\psi_2(y))^n \\ &= -\delta_R - n\delta_R \left(\frac{\psi_2(y)}{\bar{q}_R - \psi_2(y)}\right)^{\frac{n-1}{n}} (\bar{q}_R - \psi_2(y)) - \delta_R \frac{\psi_2(y)}{\bar{q}_R - \psi_2(y)} < 0.\end{aligned}$$

□

Proposition 7. *The stability of the manifold $(d_1, d_2) = (\psi_1(y_1), \psi_2(y_2))$ defined by setting $\epsilon = 0$ in system (21) is locally exponentially stable.*

Proof. Define $g(y_1,d_1) := -\delta_A d_1 + \delta_A (y_1 - md_1)^m (\bar{q}_A - d_1) = 0$ and $h(y_2,d_2) := -\delta_R d_2 + \delta_R (y_2 - nd_2)^n (\bar{q}_R - d_2) = 0$. The manifold $(d_1, d_2) = (\psi_1(y_1), \psi_2(y_2))$ is defined such that $g(y_1, \psi_1(y_1)) = 0$ and $h(y_2, \psi_2(y_2)) = 0$. To prove the local exponential stability of the manifold, we need to show that the

Jacobian

$$J = \begin{bmatrix} \dfrac{\partial g(y_1, d_1)}{\partial d_1} & \dfrac{\partial g(y_1, d_1)}{\partial d_2} \\ \dfrac{\partial h(y_2, d_2)}{\partial d_1} & \dfrac{\partial h(y_2, d_2)}{\partial d_2} \end{bmatrix} = \begin{bmatrix} \dfrac{\partial g(y_1, d_1)}{\partial d_1} & 0 \\ 0 & \dfrac{\partial h(y_2, d_2)}{\partial d_2} \end{bmatrix}.$$

calculated at the manifold $(d_1, d_2) = (\psi_1(y_1), \psi_2(y_2))$ has negative eigenvalues. Since this is a diagonal matrix, the problem is reduced to proving that the two following inequalities hold:

$$\begin{aligned} \left.\frac{\partial g(y_1, d_1)}{\partial d_1}\right|_{d_1=\psi_1(y_1)} &< 0 \\ \left.\frac{\partial h(y_2, d_2)}{\partial d_2}\right|_{d_2=\psi_2(y_2)} &< 0. \end{aligned} \tag{S18}$$

From the definition of the manifold,

$$0 \leq \psi_1(y_1) \leq \bar{q}_A \text{ and } 0 \leq \psi_2(y_2) \leq \bar{q}_R.$$

Additionally,

$$y_1 - \psi_1(y_1) = \sqrt[m]{\frac{\psi_1(y_1)}{\bar{q}_A - \psi_1(y_1)}} \text{ and } y_2 - \psi_2(y_2) = \sqrt[n]{\frac{\psi_2(y_2)}{\bar{q}_R - \psi_2(y_2)}}.$$

Therefore

$$\begin{aligned} \left.\frac{\partial g(y_1, d_1)}{\partial d_1}\right|_{d_1=\psi_1(y_1)} &= -\delta_A - \delta_A \left(\frac{\psi_1(y_1)}{\bar{q}_A - \psi_1(y_1)}\right)^{\frac{m-1}{m}} (\bar{q}_A - \psi_1(y_1)) - \delta_A \frac{\psi_1(y_1)}{\bar{q}_A - \psi_1(y_1)} < 0 \\ \left.\frac{\partial h(y_2, d_2)}{\partial d_2}\right|_{d_2=\psi_2(y_2)} &= -\delta_R - \delta_R \left(\frac{\psi_2(y_2)}{\bar{q}_R - \psi_2(y_2)}\right)^{\frac{n-1}{n}} (\bar{q}_R - \psi_2(y_2)) - \delta_R \frac{\psi_2(y_2)}{\bar{q}_R - \psi_2(y_2)} < 0. \end{aligned} \tag{S19}$$

□

S6 Proofs on orbital equivalence

Proposition 8. *Consider the following ordinary differential equations*

$$\dot{x} = f(x) \tag{S20}$$

$$\dot{x} = g(x) = \mu(x) f(x), \tag{S21}$$

in which $x \in \mathbb{R}^n$*,* $f : \mathbb{R}^n \to \mathbb{R}^n$ *is Lipschitz continuous and* $0 < a \leq \mu(x) \leq b < \infty$ *is a Lipschitz continuous scalar function. Then, there exists a function* $\alpha : \mathbb{R} \to \mathbb{R}$*, monotonically increasing and bounded such that if* $\phi(t)$*,* $t \in \mathbb{R}^n$ *is a solution of (S20) with initial condition* $x = x_0$*, then* $\psi(t) := \phi(\alpha(t))$*, is a solution of (S21) with the same initial conditions. Furthermore,* $\dfrac{d\alpha(t)}{dt} = \mu(\phi(\alpha(t)))$*.*

Proof. Since $\phi(t)$ is a solution of (S20), for all $t > 0$, we have that $\dfrac{d\phi(t)}{dt} = f(\phi(t))$. Let $\alpha(t)$ be the solution of the ordinary differential equation

$$\frac{d\alpha}{dt} = \mu(\phi(\alpha)) \tag{S22}$$

with initial condition $\alpha(0) = 0$. Let also $\psi(t)$ be defined as above. Since $g(x)$ is Lipschitz continuous, system (S21) has an unique local solution at the point $\psi(t)$ whose tangent is given by $g(\psi(t))$. The vector tangent to $\psi(t)$ is given by

$$\frac{d\psi(t)}{dt} = \frac{d\phi(\alpha(t))}{dt} = \frac{d\phi(\alpha)}{d\alpha}\frac{d\alpha(t)}{dt} = f(\psi(t))\mu(\psi(t)) = g(\psi(t)) \tag{S23}$$

for all t. Additionally, note that $\alpha(0) = 0$ and therefore $\psi(0) = \phi(0) = x_0$. It follows that $\psi(t)$ is the solution for (S21) with initial condition $x = x_0$. □

The following proposition is used to show that the addition of load will increase the period.

Proposition 9. *Consider the ordinary differential equations (S20-S21) under the same conditions as in Proposition 8. Assume that (S20) has a periodic solution* $\phi(t)$ *with period* T*. If* $\mu(x) < 1$*, then the solution of (S21) is a periodic solution with period* $T' > T$*.*

Proof. From Proposition 8, we have that $\psi(t) := \phi(\alpha(t))$ is a solution for (S21), in which $\alpha(t)$ satisfies

the differential equation

$$\frac{d\alpha(t)}{dt} = \mu(\phi(\alpha(t))). \tag{S24}$$

Since the solution $\alpha(t)$ is monotonic and unbounded and since $\alpha(0) = 0$, for all $T > 0$, there is $T' > 0$ such that $\alpha(T) = T'$. Since $\phi(T) = \phi(0)$, $\psi(T') = \psi(0)$, and hence ψ is periodic with period T'. From (S24) and the fact that $\mu(x) < 1$,

$$T' = \alpha(T) = \int_0^T \mu(\phi(\alpha(t)))dt < \int_0^T 1dt = T. \tag{S25}$$

□

S7 Mechanistic Model for Stochastic Simulation

For the analysis employing the stochastic simulation algorithm [27], we considered a mechanistic model that includes all the reactions in Table S1. Table S2 gives the description the states.

This system is equivalent to the system 20 with $m = n = 2$. We consider a one-step model for protein expression and assume the rate of expression is a function of whether the promoter p_A and p_R are free, bound to an activator dimer and bound to a repressor dimer in the case of p_A. Additionally, we consider the dynamics of the dimerization of both transcription factors.

The degradation rate δ_R was the parameter chosen to generate a model for a functioning and a non-functioning clock. The total number of promoters in both simulations was $p_{A,T} = p_{R,T} = 5$. Changes in the number of binding sites q_A and q_R were used to generate retroactivity to the activator and repressor respectively.

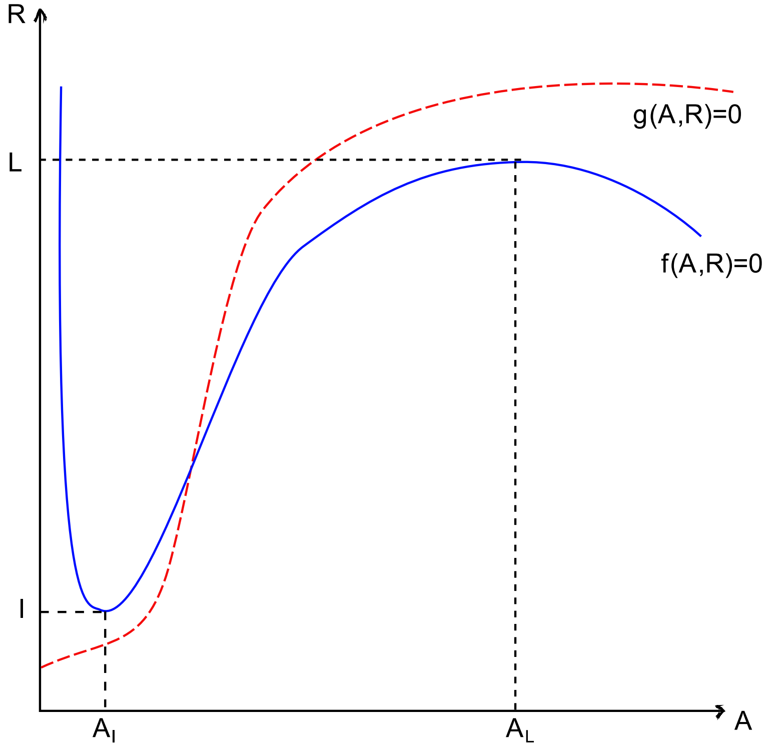

Figure S1. Nullclines and the values A_L, A_l, L, and l.

Table S1. Reactions considered in the mechanistic model

Reaction	Description	Rate	Value
$2R \to R_2$	Repressor Dimerization	k_{ra}	200
$R_2 \to 2R$	Repressor Monomerization	k_{rb}	200
$2A \to A_2$	Activator Dimerization	k_{aa}	200
$A_2 \to 2A$	Activator Monomerization	k_{ab}	200
$p_R + A_2 \to C_3$	Activator Binding	k_{a1}	2000
$C_3 \to p_R + A_2$	Activator Dissociation	k_{b1}	2000
$C_3 \to C_3 + R$	Repressor Maximal Expression	κ_3	100
$p_R \to p_R + R$	Repressor Basal Expression	κ_4	.004
$p_A + A_2 \to C_1$	Activator Binding	k_{a1}	2000
$C_1 \to p_A + A_2$	Activator Dissociation	k_{b1}	2000
$p_A + R_2 \to C_2$	Repressor Binding	k_{a2}	2000
$C_2 \to p_A + R_2$	Repressor Dissociation	k_{b2}	2000
$C_1 \to C_1 + A$	Activator Maximal Expression	κ_1	100
$p_A \to p_A + A_2$	Activator Basal Expression	κ_2	.04
$A \to \emptyset$	Activator Monomer Degradation	δ_A	1
$R \to \emptyset$	Repressor Monomer Degradation	δ_R	.2 / .4
$A_2 \to \emptyset$	Activator Dimer Degradation	δ_A	1
$R_2 \to \emptyset$	Repressor Dimer Degradation	δ_R	.2 /.4
$q_A + A_2 \to D_1$	Activator-Load Binding	k_{a1}	2000
$D_1 \to q_A + A_2$	Activator-Load Dissociation	k_{b1}	2000
$q_R + R_2 \to D_2$	Repressor-Load Binding	k_{a1}	2000
$D_2 \to q_R + R_2$	Repressor-Load Dissociation	k_{b1}	2000

Table S2. Species in mechanistic model

State	Species
R	Repressor Monomer
R_2	Repressor Dimer
A	Activator Monomer
A_2	Activator Dimer
p_R	Promoter Regulating Repressor Expression
p_A	Promoter Regulating Activator Expression
C_1	Promoter-Activator Complex, Activator Expression
C_2	Promoter-Repressor Complex, Activator Expression
C_3	Promoter-Activator Complex, Repressor Expression
q_A	Load with affinity to the activator
q_R	Load with affinity to the repressor
D_1	Activator-Load Complex
D_2	Repressor-Load Complex
